# Decreased expression of LATS1 is correlated with the progression and prognosis of glioma

**DOI:** 10.1186/1756-9966-31-67

**Published:** 2012-08-21

**Authors:** Tianhai Ji, Dan Liu, Wei Shao, Wensheng Yang, Haiqiao Wu, Xiuwu Bian

**Affiliations:** 1 Institute of Pathology and Southwest Cancer Center, Southwest Hospital, Third Military Medical University, Chongqing, 400038, China; 2 Department of Pathology, Chenggong Hospital, Xiamen University, Xiamen, Fujian, 361003, China

**Keywords:** LATS1, Tumor suppressor, Prognosis, CCNA1

## Abstract

**Background:**

LATS1 is a tumor suppressor genes implicated in the pathogenesis of certain types of tumors, but its role is not known in human glioma.

**Methods:**

Using real-time PCR and immunohistochemistry, we detected the mRNA and protein expression of LATS1 in glioma. The effect of LATS1 on cell growth and invasion were investigated.

**Results:**

We found that mRNA and protein of LATS1 expression is significantly downregulated in glioma compared with normal control brain tissues. Furthermore, reduced LATS1 expression was markedly negatively correlated with WHO grade and KPS (p<0.001 and p<0.001) in glioma patients. Patients with lower LATS1 expression had a significantly shorter overall survival time than did patients with higher LATS1 expression. Multivariate analysis suggested that the level of LATS1 expression was an independent prognostic indicator (p<0.001) for the survival of patients with glioma. Forced expression of LATS1 in glioma U251 cells not only significantly suppressed cell growth, migration and invasion, but retarded cell cycle progression from G2/M to G1 in vitro. Finally, we found that overexpressed LATS1 markedly inhibited the expression level of cell cycle factor CCNA1.

**Conclusion:**

These results indicate that LATS1 is an important candidate tumor suppressor and its downregulated expression may contribute to glioma progression.

## Background

Gliomas are neuroectodermal tumors contributing to 30–45% of all human intracranial tumors that commonly arise in the white matter of cerebral hemisphere [1]. Due to its highly invasive ability, angiogenesis and the presence of necrosis surrounding brain [2,3], malignant gliomas are often incurable by surgery alone. The molecular pathogenesis of malignant gliomas is still unclear, thus a major research effort has been directed at identifying novel specific glioma-associated genes which might play significant roles in glioma carcinogenesis.

The LATS1 gene, a mammalian homolog of fly LATS originally isolated in Drosophila as a cell proliferation inhibitor [4,5], is a speculative serine/threonine kinase that localizes to the mitotic apparatus. In mammalian cells, LATS1 is phosphorylated in a cell-cycle-dependent manner and complexes with CDC2 in early mitosis. The N-terminal region of the LATS1 protein binds CDC2 to form a complex showing decreased H1 histone kinase activity, indicating a role as a negative regulator of CDC2/cyclin A [6]. Lats1- knockout mice spontaneously developed large soft tissue sarcomas and ovarian stromal cell tumors and a high sensitivity to carcinogenic treatments, suggesting that Lats1 is a tumor suppressor at least in mice [7]. The human LATS1 gene has been mapped to chromosome 6q24-25 where loss of heterozygosity has been observed in ovarian [8], cervical [9], and breast cancers [10]. Overexpressed LATS1 not only causes G2-M arrest through the inhibition of CDC2 kinase activity in breast cancer cell line in vitro [11], but also significantly inhibited the tumorigenicity in vivo by inducing apoptosis [12]. Furthermore, recent investigations demonstrated that hypermethylation of LATS1 gene promoter which caused downregulated expression of LATS1 is frequently observed in a few human tumors, such as breast cancer and astrocytoma [13,14].

Based on Takahashi et al’s report that the LATS1 gene promoter is hypermethylated in the glioma U251 cell line [13], we hypothesized that expression of LATS1 gene is decreased in glioma pathogenesis. In the present study, we examined the expression of LATS1 in gliomas and explored its role as a tumor-suppressor gene in glioma cells in vitro. We provided a preliminary molecular mechanism of LATS1-mediated cell growth suppression in glioma.

## Materials and methods

### Cell culture

Human glioma cells U251 were cultured in RPMI1640 medium (HyClone Inc, USA) supplemented with 12% new calf bovine serum (NCBS) (PAA Laboratories, Inc, Austria) in a 37°C, 5% CO_2_ incubator.

### Clinical sample collection

Samples with confirmed pathological diagnosis were collected from Chenggong Hospital, Xiamen University, China, at the time of first resections before any therapy with informed consent of all patients and approval of the ethics committee for the use of these clinical materials for research purposes. This included 17 fresh paired gliomas and adjacent normal brain tissues, 32 archived paraffin-embedded normal brain tissues and 10^3^ archived paraffin-embedded gliomas. For the use of these clinical materials for research purposes, prior written consents from the patients and approval from the Ethics Committees of our hospitals were obtained. All archived paraffin-embedded glioma samples were staged according to the 2000 glioma staging system of WHO.

### Immunohistochemistry

Paraffin sections (3 μm) from 10^3^ gliomas were deparaffinized in 100% xylene and re-hydrated in descending dilutions of ethanol and water washes. Heat-induced antigen retrieval was performed followed by blocking endogenous peroxidase activity and non-specific antigen with peroxidase blocking reagent containing 3% hydrogen peroxide and serum, respectively. Subsequently samples were incubated with goat anti-human LATS1 antibody (1:100) (Abcam, MA, USA) overnight. The sections were incubated with biotin-labeled rabbit anti-goat antibody, and subsequently incubated with streptavidin-conjugated horseradish peroxidase (HRP) (Maixin Inc, China). Sections were visualized with DAB and counterstained with hematoxylin, mounted in neutral gum, and analyzed using a bright field microscope.

### Evaluation of staining

The immunohistochemically stained tissue sections were reviewed and scored separately by two pathologists blinded to the clinical parameters. The staining intensity was scored as previously described [15]. For statistical analysis, a final staining scores of f 0–1, 2–3, 4–5, and 6–7 were respectively considered to be Negative, weak, positive and strong expression.

### Quantitative real-time PCR (qPCR)

The expression of LATS1 mRNA was measured by qPCR using SYBR Premix Ex Taq (Takara, Japan) with an Mx3000P real-time PCR system (Stratagene, La Jolla, CA, USA). For LATS1 analysis, the sequence for sense primer was 5’*- GTTAAGGGGAGAGCCAGGTCCTT-*3’, and antisense primer was 5’*- TCAAGGAAGTCCCCAGGACTGT-*3’. Parallel reactions were performed using primers (the sense primer *5’- TCATGGGTGTGAACCATGAGAA -*3’ and antisense primer *5’- GGCATGGACTGTGGTCATGAG -*3’) for GAPDH as an internal control. Comparative quantification was determined using the 2^-ΔΔCt^ method [16].

### Establishment of glioma U251 cell line stably expressing LATS1

A LATS1 cDNA clone was purchased from GeneCopoeia Incorporation. The preparation of pCDF-GFP lentiviral vectors (SBI Corporation,USA) expressing human LATS1 was performed using the following method: 1) LATS1 open reading frame(ORF) was amplified using the forward primer 5’- *CTAC*AGATCT*ATGAAGAGGAGTGAAAAGCCAGA*-3’ and the reverse primer 5’-*CAGT*AGATCT*TTAAACATATACTAGATCGCGATTT* -3’ and a BglII restriction endonuclease site was introduced; 2) LATS1 ORF digested with BglII was cloned into a BglII-digested pCDF-GFP lentivirus expression vector; 3) The LATS1 sequence was confirmed by sequence analysis. Further, the resulting lentivirus vector together with two packaging plasmids including pFIV-34 N and pVSV-G were cotransfected into 293FT cells using lipofectamine 2000 (Invitrogen, Carlsbad, CA). An “empty” vector pCDF-GFP was utilized as a negative control. After the titers were determined, the lentiviral particles were used to infect LAST-negative U251 glioma cells. Colonies with GFP expression were selected to expand culture and total RNA of all single cell clones were isolated and quantitative real-time PCR was performed to detect the mRNA level of LATS1. Each sample was measured at least three times.

### Western blot analysis

Approximately 5 × 10^6^ U251 cells were lysed in RIPA Buffer and total protein concentration determined with BCA assay (Beyotime Inc, China) and 30 μg of total protein was loaded onto a 8% SDS-PAGE gel. Antibodies used for Western blot analysis included: CCNA1 (Abcam, MA, USA, 1:500), anti-ACTB antibody (Santa Cruz, USA, 1:400), and HRP-conjugated anti-rabbit secondary antibody (Zhongshan Inc, 1:2000). Each experiment was performed in triplicate.

### Cell proliferation analysis

Cell growth was determined by MTT assay (Sigma, USA). Briefly, 1 × 10^3^ cells were seeded into 96-well plate with quadruplicate for each condition. MTT reagent was added to each well at 5 mg/mL in 20 μL 72 h later and incubated for another 4 h. The formazan crystals formed by viable cells were then solubilized in DMSO and measured at 490 nm for the absorbance (A) values. Each experiment was performed in triplicate.

### Plate colony formation assay

Approximately 100 cells were added to each well of a six-well culture plate. After incubation at 37 °C for 15 days, cells were washed twice with PBS and stained with Giemsa solution. The number of colonies containing ≥ 50 cells was counted under a microscope [plate clone formation efficiency = (number of colonies / number of cells inoculated) × 100%]. Each experiment was performed in triplicate.

### Cell cycle analysis

The cells grown in the regular growth or the serum-free media for 36 h were collected, fixed in methanol and stained with PBS containing 10 μg/mL propidium iodide and 0.5 mg/mL RNase A for 15 min at 37 °C. The DNA content of labeled cells was acquired using FACS Caliber cytometry (BD Biosciences). Each experiment was performed in triplicate.

### Migration and invasion assay

Cells growing in the log phase were treated with trypsin and re-suspended as single-cell solution. A total of 1 × 10^5^ cells were seeded on a fibronectin-coated polycarbonate membrane insert in a transwell apparatus (Corning Inc., Corning, NY). In the lower chamber, 600 μl of RPMI 1640 with 10% NBCS was added as chemoattractant. After the cells were incubated for 18 h, the insert was washed with PBS, and cells on the top surface of the insert were removed by a cotton swab. Cells adhering to the lower surface were fixed with methanol, stained with Giemsa and counted under a microscope in five predetermined fields (×100). All assays were independently repeated at least three times. For the matrigel invasion assay, the procedure was similar to the cell migration assay, except transwell membranes were precoated with 25 μg/μl Matrigel (R&D Systems, USA). The cells were incubated for 18 hours at 37 °C and 5% CO_2_ incubator_._ Cells adhering to the lower surface were fixed by methanol, stained by Giemsa and counted under a microscope in five predetermined fields (×200). All assays were independently repeated at least three times.

### Statistical analyses

All statistical analyses were performed using SPSS 13.0 software. The χ^2^ test was used to analyze the correlation between the levels of LATS1 expression and clinicopathologic characteristics. Survival curves were plotted using the Kaplan-Meier method and compared using the log-rank test. The significances of various variables in survival were analyzed using Multivariate Cox Proportional Hazards Model. One-way ANOVA was used to determine the differences between groups for all in vitro analyses. A *P* value of less than 0.05 was considered statistically significant.

## Results

### Downregulated mRNA expression of LATS1 in Glioma

**Figure 1:**
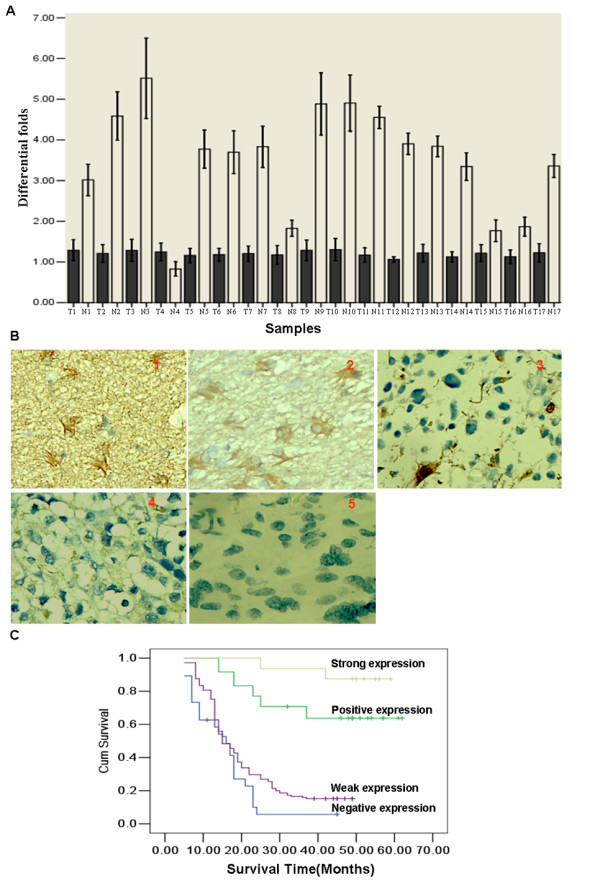
**The reduced expression levels of LATS1 mRNA and protein in glioma and Kaplan–Meier plots of overall survival duration in patients with glioma. A**. LATS1 mRNA level was markedly downregulated in glioma samples compared to their matched normal brain tissues. **B**. Reduced protein expression of LATS1 in glioma. 1: Strong expression of LATS1 in normal brain; **2:** Strong expression of LATS1 in glioma WHO grade-1; **3:** Strong expression of LATS1 in glioma WHO grade-2; **4:** Weak expression of LATS1 in glioma WHO grade-3. **5.** Negative expression of LATS1 in glioma WHO grade-4; **C**. Kaplan–Meier survival analysis of overall survival duration in 103 glioma patients according to LATS1 protein expression. The log-rank test was used to calculate *p* values.

In order to assess the role of LATS1 in glioma, we performed real-time PCR to measure the expression of LATS1 mRNA transcripts in 17 paired gliomas and their adjacent brain tissues. As shown in Figure 1A, 13 glioma tissues showed the markedly decreased expression (>2-fold change) of LATS1 compared to their matched normal tissues (Figure 1A).

### Reduced LATS1 protein expression in glioma

We measured the expression levels and subcellular localization of LATS1 protein in archived paraffin-embedded normal brain and glioma samples using immunohistochemical staining (Figure 1B1-B5). LATS1 protein is primarily localized within the cytoplasm. Furthermore, we observed expression of LATS1 was markedly decreased in glioma samples compared to normal brain tissues (p<0.001) (Table 1).

**Table 1:** The expression of LATS1 protein in Glioma and normal brain

**Group**		**Expression Level of LATS1 Protein(n)**				**P**
	**Cases (n)**	**Negative**	**Weak**	**Positive**	**Strong**	
Glioma	103	23	52	20	8	
Normal brain	32	1	3	12	16	P<0.001

### Relationship between clinicopathologic features and LATS1 expression in glioma patients

The relationships between clinicopathologic features and LATS1 expression levels in individuals with glioma were analyzed. We did not find a significant association of LATS1 expression levels with patient’s age and sex in 10^3^ glioma cases. However, we observed that the expression level of LATS1 was negatively correlated with WHO grade (P<0.016) and KPS in glioma patients (Table 2).

**Table 2:** The correlation of LATS1 protein expression with Clinicopathological features in Glioma

**Clinicopathological features**	**Cases (n)**	**Expression Level of LATS1 Protein(n)**				**P**
		**Negative**	**Weak**	**Positive**	**Strong**	
Age						
≥55	47	11	22	9	5	
< 55	56	12	30	11	3	P = 0.752
Gender						
Male	60	13	35	7	5	
Female	43	10	17	13	3	P = 0.326
WHO grade						
I	19	1	6	8	4	
II	22	3	11	6	2	
III	30	7	19	3	1	
IV	32	12	16	3	1	P<0.001
KPS						
≥80	53	6	28	13	6	
<80	50	14	24	7	2	P = 0.011

### Survival analysis

**Figure 2:**
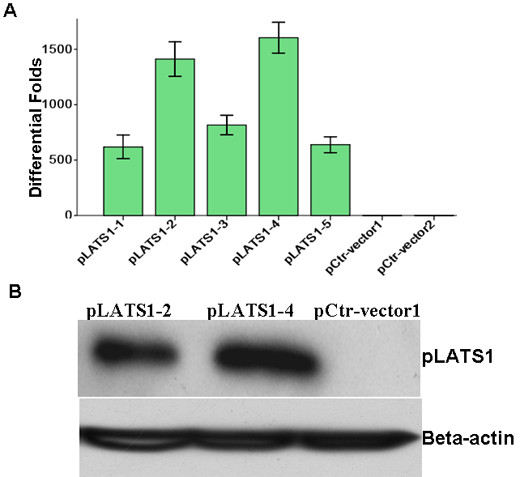
**Reexpression of LATS1 in glioma U251 cells.A**. Real-time PCR analysis indicated the highest mRNA expression of LATS1 in two cell clones pLATS1-2 and −4. **B**. Western blotting assay shows significantly increased protein expression of *LATS1* in pLATS1-2 and −4 suppressed the expression of cell cycle factor CCNA1 protein compared to Control-vector cells. β-actin was used as the internal control.

To investigate the prognostic value of LATS1 expression for glioma, we assessed the association between levels of LATS1 expression and patients’ survival using Kaplan–Meier analysis with the log-rank test. In 10^3^ glioma cases with prognosis information, we observed that the level of LATS1 protein expression was significantly correlated with the overall survival of glioma patients (Figure 2C). Patients with negative and weak level of LATS1 expression had poorer survival than those with positive and strong level of LATS1 expression (*P*<0.001). In addition, WHO grade and KPS were also significantly correlated with patients’ survival (*P*<0.001 and *P*<0.001 respectively). To determine whether LATS1 expression is an independent prognostic factor for glioma, we performed multivariate analysis of the levels of LATS1 protein expression adjusted for LATS1 expression, WHO grade, and KPS of glioma patients. The results showed that the level of LATS1 expression was an independent prognostic factor for glioma (*P*<0.001) (Table 3).

**Table 3:** Summary of univariate and multivariate Cox regression analysis of overall survival duration

**Parameter**	**Univariate analysis**			**Multivariate analysis**		
	***P***	**HR**	**95%CI**	***P***	**HR**	**95%CI**
Age						
≥55vs. <55 years	0.069	0.777	0.593-1.019			
Gender						
Male vs. female	0.160	0.820	0.621-1.082			
WHO grade						
Ivs.II vs.III vs.IV	0.000	1.715	1.454-2.023	0.000	1.463	1.233-1.735
KPS						
≥80 vs. < 80	0.000	2.033	1.540-2.684	0.000	2.437	1.810-3.283
LAST1 expression						
Strong vs.Positive vs.Weak vs.Negative*	0.000	0.437	0.362-0.528	0.000	0.389	0.316-0.478

### Overexpression of LATS1 in glioma U251 cells

To study its biological functions, we introduced the LATS1 gene into the glioma U251 cell line using pCDF-GFP lentivirus expression vector. Five (5) stably transfected cell clones were obtained. Real-time PCR identified two cell clones (LATS1-2,-4) with the highest mRNA expression of LATS1 (Figure 2A). Further, LATS1 protein was highly expressed in two cell clones by western blotting assay with LATS1 antibody,while control clone cells lacked similar expression (Figure 2B).

### LATS1 inhibits cell proliferation *in vitro*

**Figure 3:**
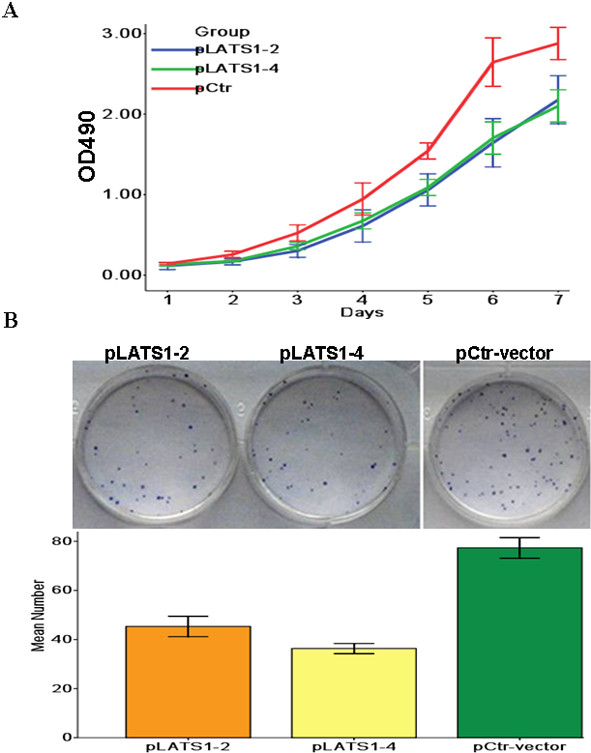
**Overexpression of LATS1 inhibted cell proliferation in vitro.A**. The cell growth of Control-vector cells and pLATS1-2 and −4 cells, were examined by MTT assay over a seven-day period. *P < 0.05, as compared to control-vector cells. **B**. The cell growth of control-vector cells and pLATS1-2 and −4 cells, were examined by plate colony formation assay. *P < 0.05, as compared to control-vector cells.

To analyze the function of LATS1, we studied the rate of cell proliferation of LATS1-expressing LATS1-2 and −4 cells. The growth curves determined by MTT assay revealed that LATS1 significantly inhibited cell proliferation of these two lines of cells compared to control clone cells (Figure 3A). In a colony formation assay LATS1-overexpressing LATS1-2 and −4 cells formed significantly less colonies than control clone cells (*P* < 0.001 for both cell types) (Figure 3B, Table 4), suggesting the inhibitory effect of LATS1 on anchorage-dependent growth of glioma cells.

**Table 4:** Plate clone formation assay among pLATS1-2, pLATS1-4, and Ctr-vector cells

**Cells**	**Number**	**P value**
pLATS1-2	45.33 ± 4.16	
pLATS1-4	34.67 ± 6.25	
Ctr-vector	77.33 ± 7.12	p<0.001

### LATS1 suppressed cell migration and invasion

**Figure 4:**
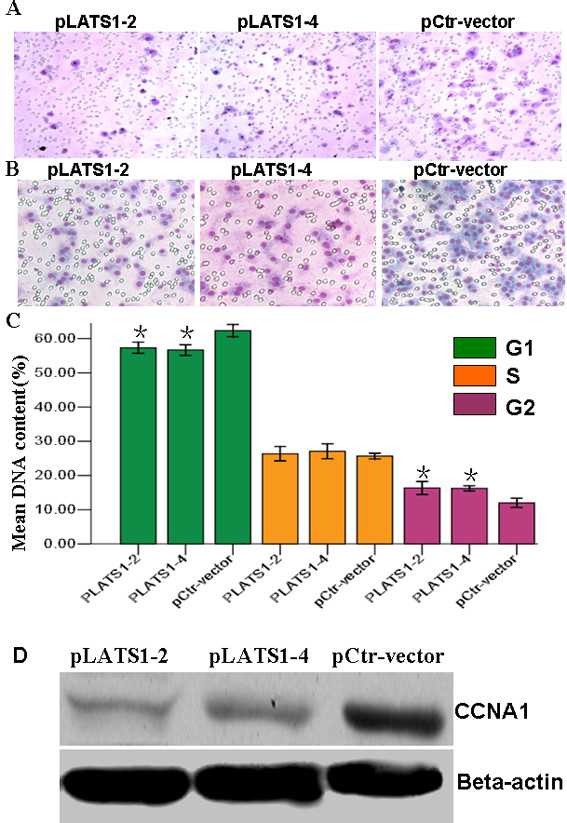
**Increased LATS1 expression inhibited cell migration, invasion and cell cycle progression.** (**A**) Cell migration and (**B**) invasion capabilities of pLATS1-2, -4 cells and Control-vector cells, were examined using transwell and boyden chamber assay. Data were presented as mean ± SD for three independent experiments. *P < 0.05, as compared to control-vector cells. **C**. Cell cycle in pLATS1-2 and −4 cells and control-vector cells, was determined by FACS Caliber Cytometry. *P < 0.05, as compared to control-vector cells.

We employed the *in vitro* migration assay to assess the migration ability of LATS1-overexpressing U251 cells. The migration of LATS1-overexpressing LATS1-2 and −4 cells was significantly slower than that of the control cells (Figure 4A). Using a boyden chamber coated with matrigel, we determined changes in cell invasiveness after 18-h incubation. Compared with the negative control cells, LATS1-expressing −2 and −4 cells both showed significantly decreased invasiveness (for both *P* < 0.001) (Figure 4B).

### Inhibition of cell cycle progression by LATS1

To detect the effect of *LATS1* on cell cycle, we measured cell cycle distribution in LATS1-expressing −2 and −4 cells. The G2 phase population was markedly increased and G1 phase population significantly decreased in both cell lines compared to the Ctr-vector cells and U251 cells (*P* < 0.001). However, in both two lines the change in S phase population was not significant (Figure 4C)(Additional file 1: Figure S1)(Additional file 2: Table S1).

### LATS1 inhibits the expression of CCNA1

In exploring the molecular mechanism of LATS1 tumor-suppressing function in glioma, we found that restoration of LATS1 expression significantly inhibited expression of cell cycle factor CCNA1 in glioma U251 cells (Figure 4D). This suggested that LATS1 may be involved in G2/M cell cycle pathway in glioma.

## Discussion

Malignant gliomas occur more frequently than other types of primary CNS tumors, having a combined incidence of 5–8/100,000 population. Due to its highly invasive nature, median reported survival is less than 1 year even with aggressive treatment using surgery, radiation, and chemotherapy [17]. Thus, there is a need for a better understanding of the molecular basis of glioma pathogenesis to improve prognosis prediction and develop targeted, molecular-based therapies.

Accumulating evidence suggests that the LATS (Large Tumor Suppressor) family of human tumor suppressors (LATS1 and LATS2) as regulators of cellular homeostasis. Loss of function of either LATS1 or LATS2 leads to a variety of tumor types including soft tissue sarcomas, leukemia, as well as breast, prostate, lung and esophageal cancers [18], which suggests they function as tumor suppressors in tumor pathogenesis. LATS1 gene is located at chromosome 6q25.1 and its open reading frame is 3393 bp encoding a 1130-amino acid polypeptide with molecular weight of 126.87 kDa. LATS1 expression is significantly decreased in some tumors including breast cancer and astrocytoma [13,14], and this downregulation has been attributed to its promoter hypermethylation. Negative regulator of oncoprotein YAP1 in the Hippo signaling pathway plays a pivotal role in organ size control and tumor suppression by restricting proliferation and promoting apoptosis. LATS1 phosphorylates YAP1 protein and inhibits its translocation into the nucleus to regulate cellular genes important for cell proliferation, cell death, and cell migration [19]. Furthermore, in previous studies LATS1 overexpression induced cell apoptosis by increasing pro-apoptotic proteins p53 and Bax [11] and suppressed cell proliferation through p53 upregulation to ensure genomic integrity [20]. Conversely, knockdown of LATS1 induced cell migration in HeLa cells [21]. These results together supported that LATS1 played a suppressive role in tumor pathogenesis.

In order to assess the role of LATS1 in glioma, we first performed real-time PCR to measure the expression of LATS1 mRNA transcripts in 17 paired glioma samples and their adjacent brain tissues. Similar to reports of other tumor types [13,14], we observed that LATS1 expression was significantly decreased in 13 glioma tissues compared to their matched normal tissues. This suggested LATS1 functions as a tumor suppressor in glioma. We validated this downregulation of LATS1 protein by immunohistochemistry. In addition, we found that LATS1 expression levels were inversely associated with WHO grade of glioma and KPS. Further, we presented the evidence that LATS1 protein expression in glioma was positively correlated with patient’s overall survival. The patients with lower expression of LATS1 protein had shorter survival time. According to multivariate analyses, decreased expression of LATS1 protein was a significant predictor of poor prognosis for glioma patients. These results were analogous to Takahashi et al’s report in the study of breast cancer [13] and strongly suggested a suppressive role of LATS1 in glioma tumorigenesis.

Next, we used a gain-of-function approach by introducing the LATS1 gene into LATS1-negative U251 glioma cells, to investigate its biological functions. We observed that overexpression of LATS1 caused significant reduced *in vitro* cell growth and G(2)/M arrest. These are consistent with the findings by Yang et al. [11] and Xia *et al.*[12] that upregulation of LATS1 suppresses cell growth and cell cycle progression, which further demonstrates that the suppressive biological functions of LATS1 are common to multiple cancers. Additionally, our study also revealed a novel function of LATS1 in glioma in suppression of cell migration and invasion. This suggests LATS1 may be involved in invasion and metastasis of cancer, a concept which would need to be confirmed by *in vivo* animal model. The observations that LATS1 regulates multiple cellular processes such as cell proliferation, cell cycle progression, migration, invasion emphasizes its importance as a therapeutic target for treating glioma.

In a previous investigation, increased LATS1 expression inhibited cell proliferation by blocking the G2/M transition, mainly through inhibition of the kinase activity of Cdc2/ Cyclin A/B complex [18]. We also observed that overexpressed LATS1 caused the G2/M phase blockade in glioma U251 cells. Therefore, we investigated the expression change of CCNA1, a cell cycle factor in the Cdc2/ Cyclin A/B complex. This gene binds both CDK2 and CDC2 kinases and thus regulates the cell cycle transition at G2/M [22-25]. We speculated CCNA1 might be involved in the cell cycle regulation pathway of LATS1 in glioma. Consistent with this presumption, we found that overexpression of LATS1 significantly reduced the expression of CCNA1 by western blot assay in glioma U251 cells. Further investigation is necessary to determine the exact role LATS1 plays in cell cycle pathway in glioma.

## Conclusions

Our results indicate that the decreased expression of LATS1 appears to favor the development of glioma and might serve a suppressive role in glioma. Further, we applied a gain-of-function approach and to examine the biological processes regulated by LATS1 in glioma cells. We demonstrated the functional importance of LATS1 in suppressing glioma cell growth, migration, invasion and cell cycle transition from G2 to M phase. Finally, we observed that overexpression of LATS1 could inhibit the expression of cell cycle factor CCNA1, which might partly explain the mechanism by which LATS1 in controls cell proliferation.

## Competing interests

The authors declare that they have no competing interests.

## Authors’ contributions

XB designed and directed the study. TJ, DL and WS performed experiments, conducted the analysis and drafted the manuscript. WY and HW assisted in the analysis and interpretation of results. All authors read and approved the final manuscript.

## Supplementary Material

Additional file 1 Figure S1.Cell cycle map of pLATS1-2, -4 cells and Control-vector cells. Click here for file

Additional file 2 **Table S1.Overexpression of LATS1 reduced DNA content of G2 phase and increased DNA content of G1 phase.** (DOC 27 kb) Click here for file
