# Supplementary material for: Decreased expression of LATS1 is correlated with the progression and prognosis of glioma
Source: J Exp Clin Cancer Res. 2012 Aug 21;31(1):67. doi: 10.1186/1756-9966-31-67 (PMC3561646; doi:10.1186/1756-9966-31-67)
Supplement: Additional file 1 — Figure S1.Cell cycle map of pLATS1-2, -4 cells and Control-vector cells. [file 1756-9966-31-67-S1.doc]

Table S1 Overexpression of LATS1 reduced DNA content of G2 phase and increased DNA content of G1 phase

| Cells | G1 | S | G2 |
| --- | --- | --- | --- |
| pLATS1-2 | 57.31±0.83 | 26.34±1.05 | 16.35±0.94 |
| pLATS1-4 | 56.68±0.78 | 27.07±1.11 | 16.25±0.38 |
| pCtr | 62.32±0.89 | 25.68±0.43 | 12.00±0.70 |
