# Supplementary figures and images for: Decreased expression of LATS1 is correlated with the progression and prognosis of glioma
Source: J Exp Clin Cancer Res. 2012 Aug 21;31(1):67. doi: 10.1186/1756-9966-31-67 (PMC3561646; doi:10.1186/1756-9966-31-67)

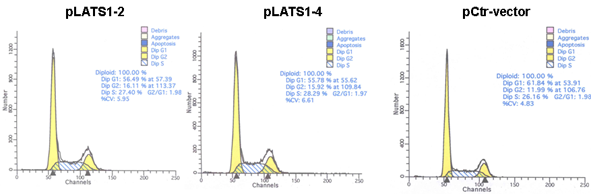

Supplement: Additional file 2 — Table S1.Overexpression of LATS1 reduced DNA content of G2 phase and increased DNA content of G1 phase. (DOC 27 kb) [file 1756-9966-31-67-S2.tiff]
